# Correlation of Uric Acid with Glomerular Filtration Rate in Chronic Kidney Disease

**DOI:** 10.31729/jnma.3700

**Published:** 2018-08-31

**Authors:** Milan Khadka, Binod Pantha, Lochan Karki

**Affiliations:** 1 Department of Medicine, NAMS, Bir Hospital, Kathmandu, Nepal

**Keywords:** *chronic kidney disease*, *estimated glomerular filtration rate*, *hyperuricemia*, *uric acid*

## Abstract

**Introduction:**

Chronic Kidney Disease is a worldwide public health problem that affects millions of people from all racial and ethnic groups. Identification of a Chronic Kidney Disease is a major risk factor for cardiovascular morbidity and mortality and is attributed to hyperuricemia. Evidences show that high serum uric acid contribute directly to glomerulosclerosis, interstitial fibrosis and atherosclerosis that correction of hyperuricemia associated with Chronic Kidney Disease will slow the progression of chronic renal failure. This study is done to correlate between serum uric acid level and estimated glomerular filtration rate in chronic kidney disease patients.

**Methods:**

A hospital based cross-sectional study on chronic kidney disease patients including 57 patients on conservative treatment attending Bir Hospital with diagnosis of chronic kidney disease was performed. Detailed clinical history, examination and investigations including uric acid were done. Chronic Kidney Disease staging was done according to estimated glomerular filtration rate estimated by Cockcroft-Gault equation. Prevalence rate of hyperuricemia in Chronic Kidney Disease and its stages were calculated and compared with each other.

**Results:**

A total of 57 Chronic Kidney Disease cases were enrolled, with male to female ratio of 2:1 and mean age 51.63±17.75 years. Hyperuricemia was present in 55 (96.49%) of study population. Though prevalence of hyperuricemia increased with Chronic Kidney Disease stage, there was no significant difference in mean value of uric acid in different stages. Hyperuricemia and stages of Chronic Kidney Disease had negative correlation which was statistically significant.

**Conclusions:**

Hyperuricemia is highly prevalent among Chronic Kidney Disease patients with conservative management. The severity of hyperuricemia increases as Chronic Kidney Disease stage progresses.

## INTRODUCTION

Chronic Kidney Disease (CKD) is defined as abnormalities of kidney structure or function, present for more than 3 months, with implications for health. Kidney damage refers to a broad range of abnormalities of kidney resulting in decline of excretory, endocrine and metabolic functions. KDIGO refers to a eGFR<60 ml/min/1.73m^2^ as decreased eGFRand eGFR<15 ml/ min/1.73m^2^ as kidney failure.^1^ Hyperuricemia is defined as serum uric acid more than 7 mg/dl in males and 6 mg/dl in females.^2^

Chronic hyperuricemia is strongly associated with chronic tubulointerstital disease and decreased renal function.^3,4^ Evidence reported on an association of uric acid with hypertension and renal disease.^5^ It is a potent antioxidant outside cell, pro-oxidant inside cell and induce stimulation of NADPH oxidases with mitochondrial dysfunction.^6^ It leads to arteriosclerosis, glomerular hypertension and glomerulosclerosis and progress CKD.^7^

This study is done to know the prevalence of high serum uric acid level in chronic renal disease and to correlate between severity of the renal disease and serum uric acid level.

## METHODS

It is a hospital based cross-sectional study. It comprises 57 patients with CKD on conservative management who attended Department of Nephrology, Bir Hospital from May 2015 to April 2016. The study was performed after approval from the Institutional Review Board at our hospital and taking written, informed consent from all the patients. All cases of CKD stage- 3,4,5 who were on conservative management were included. Excluded cases were: 1. Renal transplant patients, 2. Patients on uric acid lowering drugs, 3. CKD patients on hemodialysis and 4. Patients not willing to give consent.

Non-random sampling technique was applied. Patients admitted and attending outpatient department in hospital with clinical, biochemical and sonographic evidence of CKD were taken. Sample size was calculated using following formula:


$$\begin{array}{l} \text{n = }{\text{Zα}}^{2}{\text{PQ/d}}^{2}\\ \text{n = }57 \end{array}$$

Where

n= required sample size
Za= z deviated corresponding to desired reliability level (1.96)
P= estimated proportion in the population (10.6%)^8^
Q= 100-P (if P is in %)
d = maximum tolerable error (10%)

CKD staging is done as following (Table 1 ). A detailed clinical history was recorded from the patients in the predesigned proforma. Data relating to demographic profile including age, gender, residence, occupation were recorded. Presenting features along with duration, history of smoking, alcohol consumption significant past history of diabetes mellitus (DM), hypertension and treatment were recorded. A thorough clinical examination was done and vitals- blood pressure and pulse rate, height, weight and relevant systemic examination. Then routine laboratory tests for CKD such as complete blood count, blood sugar, urea, creatinine, sodium, potassium, calcium, phosphorus, total protein, albumin, urine analysis, abdominal ultrasonogram, and electrocardiogram were performed and after confirming diagnosis of CKD with staging, blood sample for UA was sent.

**Table 1:** CKD staging based on estimated GFR.

Stage (GFR Category)		eGFR, mL/min per 1.73 m^2^	Terms
1		≥90	Normal or high
2		60–89	Mildly decreased
3	a	45–59	Mildly to moderately decreased
	b	30–44	Moderately to severely decreased
4		15–29	Severely decreased
5		<15	Kidney failure

Data was tabulated and interim analysis was performed after the reports of 10 cases are available. All the analysis were carried out using standard statistical software SPSS 20 version and Microsoft Excel. Results are presented in tables, graphs and diagrams. Results were expressed as mean±SD. Prevalence rate of hyperuricemia in CKD and its stages were calculated and compared with each other. Statistical analyses included the unpaired t-test (for continuous measures) and the Chi-square tests for categorical assessed. For the purpose of this study a 95% confidence interval was accepted and a P<0.05 was taken as significant.

## RESULTS

**Figure 1.**
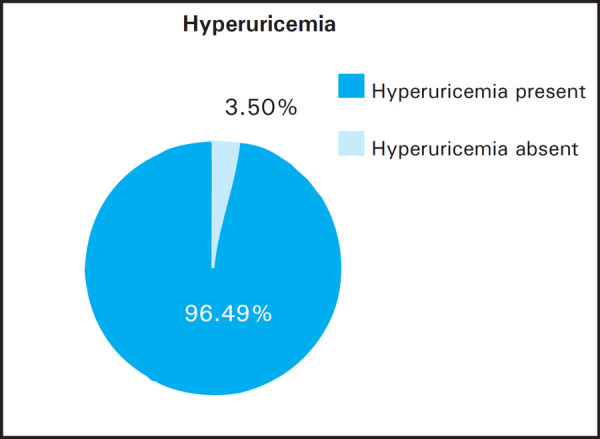
Hyperuricemia in study population.

Out of 57 patients, 38 were male and 19 were female. The mean age for the total number of patients was 51.63 years. Hyperuricemia was present in 55 (96.49%) of the patients (Figure 1).

**Figure 2.**
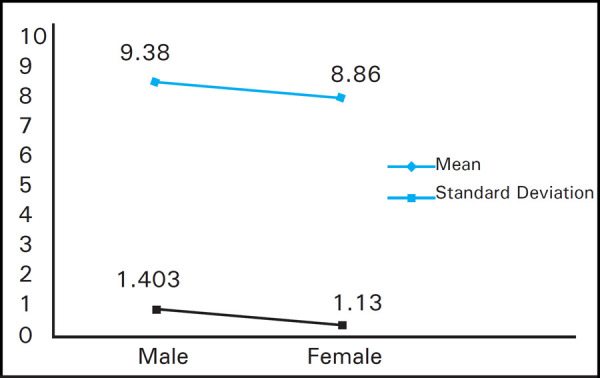
Hyperuricemia gender cross tabulation.

The mean uric acid level in men was 9.38±1.403 mg/ dl and mean uric acid level in female population was 8.86±1.13 mg/dl (Figure 2).

**Figure 3.**
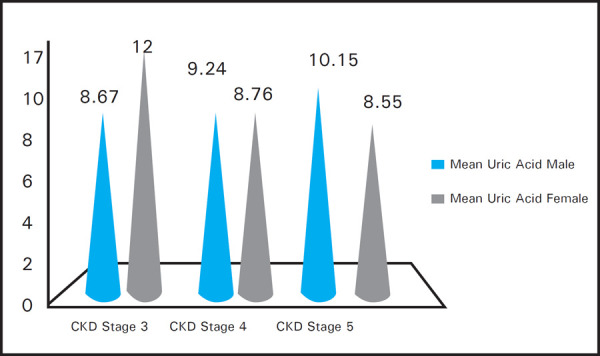
Hyperuricemia in various stages of CKD.

The mean uric acid level in stage 3 CKD in female population was 12 mg/dl and in men population was 8.67 mg/dl. In stage 4 CKD mean uric acid level in female was 8.76 mg/dl and in men was 9.24 mg/dl and in stage 5 CKD mean uric acid level in female was 8.55 mg/dl and in men was 10.15 mg/dl (Figure 3).

**Figure 4.**
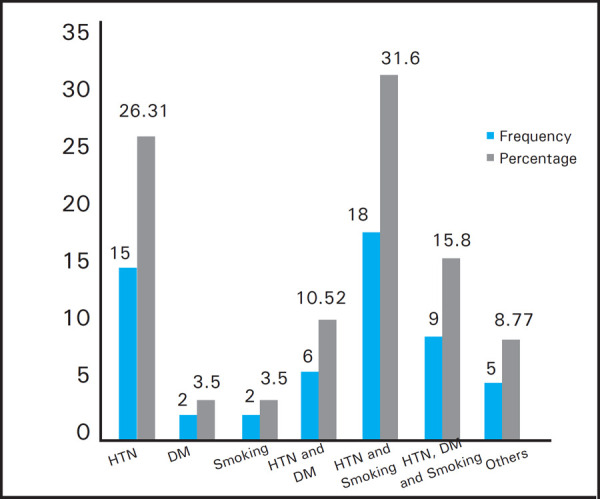
Causes of CKD according to study population.

Of the study population 15 (26.31%) had hypertension, 6 (10.52%) had hypertension and DM, 18 (31.6%) had hypertension and smoking, 2 (3.5%) had DM, 9 (15.8%) had hypertension, DM and smoking and 2 (3.5%) were only smokers (Figure 4).

## DISCUSSION

This study has evaluated prevalence of hyperuricemia in Chronic Kidney Disease. Patients in a cohort of 57 subjects, out of which 38 patients (66.7%) were male and 19 patients (33.3%) were females. In this study, hyperuricemia was present in 96.49% of study population while 3.51% has normal serum uric acid. The mean serum uric acid in the study population was 9.213 mg/dl. Similar study done by Jolly ES et al. in which a 1078 participants was analysed in Genetics of Coronary Artery Disease in Alaska Natives (GOCADAN) study in which the participants were followed for 4 years. Sixty three (13%) of the participants had CKD. The mean age of the participants having CKD was 63 years. Forty seven (63%) of them had HTN and 7 (9%) had DM. BMI of the CKD patients was 28.5 kg/m^2^. The mean serum uric acid of the study participants was 6.5±1.9 mg/dl.^9^

In the study done by Paudel YP et al. at Kist Medical College and Teaching Hospital in Kathmandu involving 150 CKD patients, found 57% male, 43% female, mean age was 48.62±18.09 years. The mean uric acid level was 5.96±2.22mg/dl. In this study, the prevalence of hyperuricemia in various stage was CKD stage 3–24.6%, stage 4–40.4% and stage 5–35% study population. There is no significant difference in the mean value of serum uric acid in the various stages of CKD.^10^

Similar study done by Adejumo OA et al, prevalence of hyperuricemia in stage 3 CKD patients was 33.4%, stage 4–40.8%and stage 5–21.7%. In this study, mean creatinine level of the patient was 4.1298 mg/ dl. The mean systolic BP was 156.3860 mm of Hg and mean diastolic BP was 93.7544 mm of Hg. Out of 120 patients, 57 of the patients had high serum uric acid level. The prevalence of hyperuricemia was 47.5%.^11^

Most of the patients in our study were hypertensive, so it can be concluded that hyperuricemia may lead to the progession of CKD faster in hypertensive patients. Study done by Sedaghat S et al. in a prospective cohort showed that high levels of serum uric acid are associated with faster decline in eGFR and increased incidence of CKD.^12^ The association was more pronounced in hypertensive subjects compared to normotensive individuals. In this study the most common cause for CKD was hypertension 57.89% of studied population had HTN. Hypertension and DM both were present on 27.31% of the patient. DM alone was present on 3.5% of the patients. The remaining 12.28% of the patient had other causes for CKD. In the study done by Adejumo OA et al. involving 120 patients the causes of CKD were that 37.8% of the patient had hypertensive nephropathy, 30% had diabetic nephropathy, 27.5% had chronic glomerulonephritis and 5.8% had other causes for CKD.

## CONCLUSIONS

This study showed that hyperuricemia is highly prevalent among CKD patients with conservative management. Though this study showed increasing prevalence of hyperuricemia with increasing stage of CKD (stage III to V), the mean value of uric acid did not show significant difference among different stages. Further studies without above mentioned limitation and including larger group are needed.

## Conflict of Interest

**None.**
